# Conformational alterations in the protein-DNA complex facilitates efficient integration of diverse DNA substrates by the CRISPR Cas1-Cas2 proteins

**DOI:** 10.1063/4.0001082

**Published:** 2025-10-27

**Authors:** Alberto Monteiro Dos Santos, Saadi Rostami, Richard Van, Kole Long, Swarmistha Devi Aribam, Yihan Shao, Rakhi Rajan

**Affiliations:** University of Oklahoma, Norman, OK, USA

## Abstract

CRISPR-Cas [clustered regularly interspaced short palindromic repeats (CRISPR)-CRISPR-associated] systems are adaptive immune systems present in bacteria and archaea to fight invading genetic elements. While proteins such as Cas9 and Cas12a cleave and inactivate the invader DNA, the mechanisms of which have been repurposed into efficient gene editing and gene therapy tools, Cas1 and Cas2 proteins are important in creating genetic memory of past invasions of foreign elements to create an active adaptive immune mechanism. Specifically, Cas1(4)-Cas2_(2)_ complex (4:2 molar ratio of Cas1 and Cas2) integrates a piece of the invader DNA called the prespacer, sequence-specifically, into the CRISPR-Cas locus. The prespacer is transcribed to form CRISPR-RNA that guides Cas9/Cas12a, sequence-specifically, to the invader on secondary infection to inactivate the invader.

While there are different types of CRISPR-Cas systems, Cas1-Cas2 proteins belonging to the type II-A CRISPR-Cas systems are unique since they can sequence-specifically integrate DNA into the CRISPR-Cas locus without additional protein factors, which is the case for other CRISPR types. This provides a unique opportunity to develop type II-A Cas1-Cas2 proteins as a site-specific integration tool for biotechnology and molecular biology applications. Previous studies from our lab have demonstrated that there are three different mechanisms within the type II-A systems for DNA integration that is based on protein-DNA sequence conservation and interactions. Specifically, a bioinformatics analysis of the “leader-repeat” junction of the CRISPR-Cas locus, a region where foreign DNA is integrated, showed three uniquely conserved junction sequences, named groups 1, 2, and 3 (G1, G2, G3). Cas1-Cas2 proteins also showed segregation mirroring that of the leader-repeat junction sequence, indicating coevolution of the DNA and protein elements. Bioinformatics results were further confirmed by *in vitro* integration assays that showed the tight dependence of the leader-repeat and protein elements of the cognate groups for successful integration. Of the different groups, G2 Cas1-Cas2 is very robust in integrating foreign DNA as an isolated system with the ability to insert diverse prespacer forms (e.g., overhang or splayed prespacer), compared to insertion of only very specific forms of the prespacer by the other two groups. This establishes G2 Cas1-Cas2 as a superior genome tool that has minimal requirements for sequence-specific DNA integration.

To gain mechanistic insights on how G2 Cas1-Cas2 proteins accommodate different prespacers, we performed molecular dynamics simulations of G2 Cas1-Cas2 proteins bound to a prespacer and a target DNA holding the leader-repeat junction. We tested two different prespacers (overhang and splayed) with two different lengths of overhang/splaying (4 vs. 5). Our results show that, of the four Cas1 monomers, two have conformational changes in the binding site that is involved in catalyzing the prespacer integration.

Specifically, two helices adopted a 3-10-helical conformation in the presence of the prespacers: one associated with the protein-DNA interaction at the leader junction and the other mediating the interactions with the divalent metal ion and the 3’-OH end of the prespacer in the active site . We propose that the presence of a 3-10-helix in key regions of active Cas1s may influence the stability of the prespacer-bound state. To understand the catalytic mechanism of DNA integration, we performed QM/MM simulations of Cas1- Cas2-prespacer-target DNA with divalent metal ions. Our results show that the number and arrangement of divalent metal ions at the active site significantly influence the structural configuration prior to catalysis. While previous research has shown a single divalent metal based catalysis, our results are hinting at the presence of a second potential Mg²^+^ binding site, suggesting a key role for metal coordination in substrate positioning, transition state stabilization, and overall enzymatic efficiency. To further dissect the reaction mechanism, we are currently investigating whether the DNA integration follows a stepwise or concerted pathway.

Other ongoing studies include cryo electron microscopy-based structure determination and analysis of protein dynamics using hydrogen- deuterium exchange mass spectrometry (HDX-MS). The end goal is to integrate insights from these complementary approaches to assess the plasticity of the Cas1-Cas2 to accommodate different DNA substrates in their active sites. This information will enable fine-tuning of their use in biotechnological applications.

